# Comparative Assessment of Serum Selenium Status in HIV-Infected and Non-infected Children: A Pilot Study in a Tertiary Hospital in Nigeria

**DOI:** 10.7759/cureus.39626

**Published:** 2023-05-29

**Authors:** Sarah O John-Olabode, Patricia Akintan, Kehinde S Okunade, Iwuchukwu Ajie

**Affiliations:** 1 Haematology and Blood Transfusion, College of Medicine, University of Lagos, Lagos, NGA; 2 Paediatrics, College of Medicine, University of Lagos, Lagos, NGA; 3 Obstetrics and Gynaecology, College of Medicine, University of Lagos, Lagos, NGA; 4 Clinical Pathology, College of Medicine, University of Lagos, Lagos, NGA

**Keywords:** pediatrics, antiretroviral therapies, cd4+ cell count, hiv, selenium deficiency

## Abstract

Background

Selenium is an essential micronutrient that plays a crucial role in a wide range of physiological processes, including immune responses. Selenium deficiency has been recognized as an associated factor in the progression of HIV to advanced HIV disease and/or mortality. Although selenium supplementation has been shown to reduce hospitalizations and improve cellular immunity, the evidence remains mixed. This study aimed to determine the prevalence of selenium deficiency and its relationship with HIV disease markers in HIV-infected children at the Lagos University Teaching Hospital.

Methodology

This is a cross-sectional, comparative, pilot study of plasma concentrations of selenium in HIV-infected (n = 30) and non-infected (n = 20) children enrolled in the pediatric HIV clinic of the Lagos University Teaching Hospital, Lagos, Nigeria, from May 2019 to May 2021. HIV-infected children were on stable antiretroviral therapy (ART) with an undetectable viral load. The serum concentration of selenium was measured using the automated atomic absorption spectrophotometer (hydride generation method). Logistic regression was used to study the effect of selenium status on the levels of HIV disease markers (CD4 count, viral load, weight, opportunistic infections) in the study participants.

Results

The median age of all participants was nine (4-12) years, with 74% being boys. The mean selenium concentrations were lower in HIV-infected children (91.1 ± 12.0 µg/L) compared to the comparison group without HIV (147.8 ± 4.9 µg/L) (p = 0.001). After controlling for age, ART duration, markers of HIV infection, and other potentially confounding variables, participants with selenium deficiency had approximately 11-fold odds of increased hospital admissions (adjusted odds ratio = 10.57, 95% confidence interval = 1.58 to 70.99; p = 0.015).

Conclusions

In this study, selenium concentrations were significantly lower in HIV-infected children than in the HIV-negative comparison group. Lower serum selenium concentrations were associated with increased hospitalizations. Although our findings suggest the potential need for selenium supplementation for children living with HIV in Nigeria, further studies are warranted to determine the safety and efficacy of selenium supplementation in this key population.

## Introduction

Despite the huge gains made in the prevention of vertical transmission of HIV, 160,000 newly acquired HIV infections were recorded globally among children (0-14 years) in 2021 [1], with 23,000 of these cases occurring in Nigeria [2]. Evidence has shown that children lag behind adults in accessing life-sustaining care, which is particularly significant in Sub-Saharan Africa where children are also nutritionally vulnerable because of poverty [2]. Micronutrient deficiency, notably selenium deficiency, has been associated with opportunistic infections, disease progression, and mortality in HIV-infected adults and children [3-6].

Selenium is an essential micronutrient that plays a crucial role in immunomodulation [7]. Low selenium concentrations are a common finding in HIV-infected individuals [8-10] and have been associated with diminished T-cell immunity. Selenium supplementation has also been found to lead to the production of a cluster of differentiation (CD)4+ T-helper (Th) cells in vitro [11].

Reliable quantification of trace elements such as selenium in biological ﬂuids is a challenging task that requires dedication and an analytical technique with high accuracy properties. Spectrometric analytical methods such as inductively coupled plasma mass spectrometry (ICP-MS), atomic ﬂuorescence spectrometry, ﬂame atomic absorption spectrometry, and electrothermal atomic absorption spectrometry are some of the recognized detection methods for trace elements. These analytical methods have their merits and demerits [12].

Animal studies have shown that selenium appears to play an important role in the virulence of microbes, including HIV. Selenium deficiency has been associated with the disruption of the oxidative mechanism of the cells involved in cellular immunity. Selenium supplementation studies have shown the potential of selenium in mitigating HIV replication and pathogenesis in vitro through the upregulation of antioxidant enzymes such as glutathione oxidase in T cells [13-15]. Selenium supplementation, therefore, appears to play a beneficial role in survival among HIV-infected individuals [6,16-19]. Campa et al., in a study of 24 HIV-infected children, showed that low plasma selenium concentration and CD4 cell count below 200/μL were independent predictors of mortality and faster disease progression [6].

However, despite findings from several observational studies showing that low serum selenium is strongly associated with HIV disease progression [6,20-23], there are still mixed reports on the effect of selenium supplementation on viral load or clinical HIV endpoints including hospitalization rate.

Although several studies have attempted to investigate the role of selenium in disease progression, morbidity, and mortality in HIV-infected adults [7-15], the role of selenium status in the HIV-infected pediatric population remains unclear due to the paucity of data. Therefore, this pilot study was designed to explore the prevalence and effects of low selenium levels on HIV disease markers in children aged 0-14 years at the Lagos University Teaching Hospital.

## Materials and methods

Study design and population

This was a cross-sectional, comparative study conducted among HIV-seropositive children who were enrolled at the pediatric HIV clinic of the Lagos University Teaching Hospital from May 2019 to May 2021. The study was approved by the hospital’s Health Research Ethics Committee (ADM/DCST/HREC/APP/3024) before the participants’ recruitment, and ethical principles according to the Helsinki Declaration were considered throughout the course of the study. Caregivers provided written informed consent, and older children above seven years old also provided informed assent. All HIV-positive participants were on stable antiretroviral therapy (ART) for at least three months with HIV-1 RNA < 50 copies/mL and were recruited during routine clinic visits. The HIV-uninfected children were recruited from the pediatric surgery outpatient clinic to serve as comparators. A previous history of acute infections (malaria, tuberculosis, and hepatitis) and diarrhea in the last three months was exclusionary. Children with known comorbidities such as diabetes and cardiovascular conditions were excluded.

Sampling techniques and data collection

As this was an exploratory pilot study designed to generate preliminary data on the effects of selenium concentrations on HIV disease markers in HIV-infected children, we adopted the minimum sample size for statistical testing within the HIV-infected children group (n = 30) and 20 HIV-uninfected children were used as comparators. Participants were selected by the consecutive sampling method over the study period, and a structured interviewer-administered questionnaire was used by the investigators to collect data from each primary parent or guardian after explaining the nature and purpose of the study. Data collected at enrollment included sociodemographic characteristics, WHO clinical staging of HIV [24], cumulative duration of ART, current CD4+ cell count, viral load, and history of hospital admissions.

Sample collection and laboratory analyses

In this study, 3 mL of non-fasting blood sample was drawn early in the morning into trace element bottles. Standard precautions for trace element determination were taken. The blood was centrifuged at 3,000 rpm for 15 minutes at room temperature, and serum was then stored at -200°C until testing. Plasma selenium concentrations were measured using the automated atomic absorption spectrophotometer (hydride generation method) (AAS Model ECI 4141, United States). Low selenium concentration was defined as selenium levels below 85 µg/L [6,9,22].

Statistical analyses

Descriptive statistics were computed for all data, and analyses were performed using the SPSS version 22.0 for Windows (IBM Corp., Armonk, NY, United States). All quantitative data were tested for normality of distribution using the Kolmogorov-Smirnov normality test. The location and spread of continuous variables were described by the mean and standard deviation (SD), respectively (if normal in distribution), or by the median and interquartile range (IQR), respectively (for skewed distribution), and the data were presented as mean ± SD or median (IQR) as appropriate. The associations between any two groups of continuous variables were tested using the independent-sample t-test (normal distribution) or the Mann-Whitney U test (skewed data), while categorical variables were tested using the chi-square test or Fisher’s exact test where appropriate. This was then followed by multivariate analyses using the binary logistic regression models at second-level analysis to study the effect of the major participants’ characteristics, including selenium status, on the levels of HIV disease markers. All significant associations were reported at p-values <0.05.

## Results

**Figure 1:**
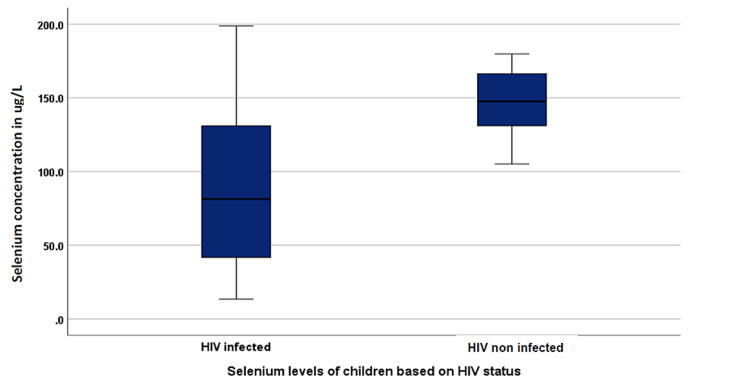
Selenium levels of participants based on HIV status (91.7 ± 12.0 vs. 147.8 ± 4.9 µg/L; p = 0.001). a: Values are presented as mean ± standard deviation.

The median age of all enrolled participants was nine (4-12) years, with 74% being boys. There was a significant difference in the mean selenium concentrations between the HIV-infected (91.1 ± 12.0 µg/L) and the HIV-negative comparison group (147.8 ± 4.9 µg/L) (Figure 1). Of the 50 participants, 16 (32%) had selenium concentration below the reference range (<85 µg/L). Notably, all 16 participants with low selenium concentrations were HIV infected, thus giving a prevalence of 53.3% for selenium deficiency among the HIV-infected children (Table 1).

**Table 1:** Characteristics of children with and without HIV. a: Values are presented as mean ± SD, median (IQR), or number (percentage) unless indicated otherwise. IQR: interquartile range; SD: standard deviation

Characteristic	Total (n = 50)	HIV (n = 30)	Non-HIV (n = 20)	P-value
Age (year)	9.0 (4.0–12.0)	10.5 (9.0–12.0)	4.0 (3.0–9.8)	0.001
Body weight (kg)	24.4 ± 11.3	30.3 ± 9.3	20.6 ± 11.6	0.002
Sex (%)				0.006
Male	37 (74.0)	18 (60.0)	19 (95.0)	
Female	13 (26.0)	12 (40.0)	1 (5.0)	
Selenium level (%)				0.001
Low (<85 µg/L)	16 (32.0)	16 (53.3)	0 (0.0)	
Normal (≥85 µg/L)	34 (68.0)	14 (46.7)	20 (100.0)	

HIV-infected participants with low and normal selenium levels did not differ significantly in terms of age (p = 0.608), viral load (p = 0.525), CD4 count (p = 0.064), WHO clinical stage (p = 0.513), and cumulative duration of ART (p = 0.400) (Table 2). However, participants with a low selenium concentration had a significantly higher rate of hospital admissions compared with participants with a normal selenium concentration (p = 0.028) (Table 1).

**Table 2:** Characteristics of HIV-infected children by selenium concentration. a: Values are presented as median (interquartile range), or number (percentage), unless indicated otherwise. ART: antiretroviral treatment

Characteristic	Total (n = 30)	Low selenium (n = 16)	Normal selenium (n = 14)	P-value
Age (year)	10.5 (9.0–12.0)	11.0 (8.3–13.0)	10.0 (9.0–12.0)	0.608
Body weight (kg)	29.8 (22.9–35.6)	30.3 (24.2–35.6)	28.2 (21.8–36.7)	0.886
ART duration (year)	8.0 (4.8–10.0)	8.5 (4.5–10.8)	7.0 (4.8–8.3)	0.400
CD4+ count (×100 cells/mm^3^)	8.2 (6.6–11.9)	10.4 (7.1–14.6)	7.2 (6.0–8.6)	0.064
Viral load (copies/mL)	20.0 (20.0–20.0)	20.0 (20.0–20.0)	20.0 (20.0–29.3)	0.525
Sex (%)				0.053
Male	18 (60.0)	7 (43.8)	11 (78.6)	
Female	12 (40.0)	9 (56.3)	3 (21.4)	
WHO clinical stage (%)				0.513
Stage 1	25 (83.3)	14 (87.5)	11 (78.6)	
Other stages	5 (16.7)	2 (12.5)	3 (21.4)	
Hospital admissions (%)				0.028
<2	15 (50.0)	5 (31.3)	10 (71.4)	
≥2	15 (50.0)	11 (68.7)	4 (28.6)	

After controlling for age, ART duration, markers of HIV infection, and other potentially confounding variables, participants with selenium deficiency had approximately 11-fold odds of increased hospital admissions (adjusted odds ratio = 10.57, 95% confidence interval = 1.58 to 70.99; p = 0.015) (Table 3).

**Table 3:** Univariate and multivariate analyses of the association between participants’ characteristics and frequent hospital admissions (more than one since the diagnosis of HIV). ART: antiretroviral treatment; CI: confidence interval; WHO: World Health Organization

Characteristics	Category	Univariate	Multivariate	
		P-value	Adjusted OR (95% CI)	P-value
Age	<11 vs. ≥11 years	0.715	-	-
Sex	Male vs. female	0.999	-	-
Weight	<30.0 vs. ≥30.0 kg	0.615	-	-
ART duration	<8 vs. ≥8 years	0.464	-	-
CD4+ count	<8.0 vs. ≥8.0 ×100 cells/mm^3^	0.269	-	-
Viral load (copies/mL)	≥20.0 vs. <20.0 copies/mL	0.143	-	-
Vitamin supplements	Yes vs. no	0.068	16.77 (1.20–23.61)	0.036
WHO clinical stage	Stage 1 vs. other stages	0.142	-	-
Selenium level	Low vs. normal	0.028	10.57 (1.58–70.99)	0.015

## Discussion

In this pilot study, we reported a relatively higher prevalence of selenium deficiency among children with HIV infection, with a significant association between selenium deficiency and increased hospitalization among HIV-infected children in Lagos, Nigeria.

The prevalence of selenium deficiency in the present cohort of HIV-infected children in Nigeria was 53.3%. This figure is higher than the prevalence of 10.9% reported by Dirajlal-Fargo et al. [23] in a similar cohort from Uganda. This difference can be explained by the difference in the analytical methods used for selenium detection in the two studies, with Dirajlal-Fargo et al. adopting the ICP-MS which is known to be more resistant to interference in comparison to the hydride generation-atomic absorption spectrometry used in this study.

Previous data have shown a significant association between HIV infection and deficiency of micronutrients such as selenium [7-9]. Indeed, nutritional deficiencies have been closely associated with immunosuppression and disease progression in HIV infection [6,10,17,20]. Factors such as virus-induced oxidative stress, malabsorption, gastrointestinal infections, and metabolic derangement have been attributed to nutritional deficiency reported in chronic HIV infection [4].

This study did not find any significant difference between HIV-infected children with and without selenium deficiency in terms of age, viral load, CD4 count, WHO clinical stage, and cumulative duration of ART. This contrasts with the report of Anyabolu et al. who found increased immunosuppression in HIV-infected children with selenium deficiency compared to HIV-infected children with normal selenium levels [4].

We reported higher hospital admissions of up to 11-fold in HIV-infected children with selenium deficiency compared to children with normal selenium concentration. This finding implies that selenium plays a significant role in reducing opportunistic infections (including tuberculosis) and improving the quality of life of HIV-infected children. This finding also corroborates previous studies that found a significant association between selenium deficiency and increased risk of tuberculosis infections, dilated cardiomyopathy, poor quality of life, and mortality in pediatric HIV infection [25-27].

To our knowledge, this is the first study that examined the association between selenium deficiency and hospital admissions among HIV-infected children in Nigeria in the post-ART era. However, these findings need to be interpreted with caution while generalizing to a larger population pending the conduct of a larger longitudinal study with a carefully calculated sample size and randomized controlled trials of selenium supplementation in HIV-infected children based on the preliminary data generated from this pilot study. Hopefully, these future studies will inform policies on the management of pediatric HIV infection in Nigeria.

The major study limitations were, first, this study was hospital-based with study participants exposed to ART therapy and virally suppressed. This may make generalizing the study findings a challenge. Second, the analytical method used to measure selenium is known to be more susceptible to interference that can serve as confounders. However, these limitations did not significantly alter the overall conclusions of this study.

## Conclusions

This study suggests the potential need for selenium supplementation in the key population of HIV-infected children in Nigeria. However, further research is recommended to determine the safety and efficacy of selenium supplementation in ART-naive and post-ART children with HIV infection with an emphasis on the benefit in HIV-associated conditions, potential drug-supplement interactions, and clinical endpoints. These findings could have implications for the long-term care of HIV in the pediatric age group.
